# Italian Dermestidae: notes on some species and an updated checklist (Coleoptera)

**DOI:** 10.3897/zookeys.360.6023

**Published:** 2013-12-06

**Authors:** Gianluca Nardi, Jiří Háva

**Affiliations:** 1 MiPAAF, Corpo Forestale dello Stato, Centro Nazionale per lo Studio e la Conservazione della Biodiversità Forestale “Bosco Fontana” di Verona, Sede di Bosco Fontana, Strada Mantova 29, I-46045 Marmirolo (MN), Italy; 2 Department of Forest Protection and Entomology, Faculty of Forestry and Wood Sciences, Czech University of Life Sciences, Kamýcká 1176, CZ-165 21, Prague 6 - Suchdol, Czech Republic

**Keywords:** Dermestidae, lectotype designation, new status, taxonomy, distribution, new records, chorotypes, checklist, Italy

## Abstract

An up-to-date checklist of the Italian Dermestidae is provided. The presence of 95 species in Italy is confirmed, while further 5 species (*Dermestes (Dermestes) vorax* Motschulsky, 1860, *Thorictuspilosus* Peyron, 1857, *T. wasmanni* Reitter, 1895, *Attagenus (Attagenus) simonis* Reitter, 1881 and *Globicornis (G.) breviclavis* (Reitter, 1878)) and 1 subspecies (*A. (A.) tigrinus pulcher* Faldermann, 1835) are excluded from the Italian fauna.

*Attagenus (Attagenus) calabricus* Reitter, 1881 and *A. (A.) lobatus* Rosenhauer, 1856 are for the first time recorded from Abruzzi and Tuscany respectively; *A. (A.) silvaticus* Zhantiev, 1976 is recorded for the first time from mainland Italy (Apulia); *Anthrenus (Anthrenus) angustefasciatus* Ganglbauer, 1904 is new to northern Italy (Friuli-Venezia Giulia), central Italy (Tuscany), Apulia and Basilicata; *A. (A.) munroi* Hinton, 1943 is new to central Italy (Elba Island); *A. (A.) delicatus* Kiesenwetter, 1851 is for the first time recorded from Apulia; *Globicornis (Globicornis) fasciata* (Fairmaire & Brisout de Barneville, 1859) is new to southern Italy (Basilicata); *G. (Hadrotoma) sulcata* (C.N.F. Brisout de Barneville, 1866) is for the first time recorded from central Italy (Abruzzi), Campania and Sicily, while*Trogoderma inclusum* LeConte, 1854 is new to Apulia.

Seven species (*Dermestes (Dermestes) peruvianus* Laporte de Castelnau, 1840, *D. (Dermestinus) carnivorus* Fabricius, 1775, *D. (Dermestinus) hankae* Háva, 1999, *D. (Dermestinus) intermedius intermedius* Kalík, 1951, *D. (Dermestinus) szekessyi* Kalík, 1950, *Anthrenus (Anthrenops) coloratus* Reitter, 1881 and *Trogodermaangustum* (Solier, 1849)) recently recorded from Italy (without further details) are discussed.

The lectotype and a paralectotype are designated for*Attagenus (A.) calabricus* Reitter, 1881 from Calabria.

*Attagenus pellio* (Linnaeus, 1758) var. *pilosissimus* Roubal, 1932 is removed from synonymy with *A. (A.) pellio* and recognized as a valid species (**stat. prom.**); it is known from Lombardy, Apulia and Calabria.

## Introduction

The main purpose of the present paper is to enhance knowledge of the Dermestidae of Italy. It is chiefly based on material collected by the staff of the Centro Nazionale per lo Studio e la Conservazione della Biodiversità Forestale “Bosco Fontana” di Verona (Italy), during entomological surveys in various Italian Protected Areas (e.g. Cerretti et al. 2003, Mason et al. 2006, Nardi and Vomero 2007, Cerretti et al. 2009, Nardi et al. 2011). Moreover, this paper gives an opportunity to provide a new checklist of the Dermestidae of Italy: it updates the nomenclature, systematics and the faunistic of the previous (Audisio et al. 1995) chiefly according to recent capital works (Háva 2007, Zhantiev 2011a, Háva 2011a).

## Material and methods

This paper is organized in three sections: “Notes on some species”, “Literature records” and “A new checklist of the Dermestidae of Italy”.

The classification of the subfamilies, tribes, genera and subgenera follows that proposed by Háva (2004, 2007); species are listed alphabetically according to the nomenclature of Háva (2007), Kadej et al. (2007), Háva (2008), Zhantiev (2009) and Háva (2011a).

The first section is based chiefly on study of new material. With very few exceptions, identifications were made by J. Háva.

The second section summarizes the literature data updating the Italian distribution of the species provided by Audisio et al. (1995) or by Háva and Nardi (2011).

In both sections, nomenclatural combinations and possible synonyms (listed chronologically-alphabetically) found in the literature that includes Italian records are provided below the valid name of each species or subspecies.

The first section concerns 25 species. As far as possible, the following data are provided for each record: region, province, general area and/or commune, locality of collection, altitude, UTM coordinates, date of collection, collector/s, collecting method, possible research project, number of specimens, and, in parentheses, abbreviation of depository. The labels of the examined specimens are generally written in Italian; hereunder, the regions and the collecting methods were translated into English. The label data of type specimens are cited verbatim between quotation marks, a forward slash separating the different lines on a label, and a comma separating subsequent labels. Comments and interpretations are given in square brackets.

The mainland Italian regions are listed from north to south, and from west to east, all toponyms are listed alphabetically. Chorotypes were assigned according to Vigna Taglianti et al. (1993, 1999) and based on the distributions provided by Háva (2003, 2007, 2011b), Zhantiev (2009, 2011a, 2011b) and Háva et al. (2013), unless otherwise stated.

In the second section, the literature records updating the checklist of Dermestidae of Italy provided by Audisio et al. (1995) or by Háva and Nardi (2011) are detailed. These new faunistic data are based on papers subsequent to these two papers but also include some previously overlooked publications. These records concern 14 species. The data on distribution are listed according to the geographic divisions by Audisio et al. (1995): northern Italy (N), peninsular Italy (S), Sardinia and small circum-Sardinian islands (Sa) and Sicily and small circum-Sicilian islands (Si).

Finally, a new checklist of the Italian Dermestidae is provided (Table 1). The total number of species for each geographic division includes only those recorded with certain, so generic records for “Italy” and records followed by a question mark are excluded.

Acronyms for chorotypes follow Vigna Taglianti et al. (1993, 1999), while those for the geographic divisions of Italy follow Audisio et al. (1995); both are included in the foreword of the checklist.

**Table 1.** Checklist of the Dermestidae of Italy.

Taxa	Distribution				Chorotype
**Dermestinae Latreille, 1804**					
**Dermestini Latreille, 1804**					
**Genus *Dermestes* Linnaeus, 1758**					
**Subgenus *Dermestes* Linnaeus, 1758**					
1. *Dermestes (Dermestes) ater* DeGeer, 1774	N				COS
2. *Dermestes (Dermestes) bicolor* Fabricius, 1781					PAL
*Dermestes (Dermestes) bicolor bicolor* Fabricius, 1781	N	S		Sa	
3. *Dermestes (Dermestes) haemorrhoidalis* Küster, 1852	N				COS
4. *Dermestes (Dermestes) hispanicus* Kalík, 1952				Sa	WME
5. *Dermestes (Dermestes) lardarius* Linnaeus, 1758	N	S	Si	Sa	COS
6. *Dermestes (Dermestes) peruvianus* Laporte de Castelnau, 1840	N?	S			COS
**Subgenus *Dermestinus* Zhantiev, 1967**					
7. *Dermestes (Dermestinus) aurichalceus* Küster, 1846	N	S?		Sa	MED
8. *Dermestes (Dermestinus) carnivorus* Fabricius, 1775		S			COS
9. *Dermestes (Dermestinus) erichsoni* Ganglbauer, 1904	N	S	Si	Sa	EUR
10. *Dermestes (Dermestinus) frischii* Kugelann, 1792	N	S	Si	Sa	COS
11. *Dermestes (Dermestinus) gyllenhalii* Laporte de Castelnau, 1840					EUR
*Dermestes (Dermestinus) gyllenhalii gyllenhalii* Laporte de Castelnau, 1840	N	S	Si?		
12. *Dermestes (Dermestinus) hankae* Háva, 1999		S			WME
13. *Dermestes (Dermestinus) intermedius* Kalík, 1951					EUR
*Dermestes (Dermestinus) intermedius intermedius* Kalík, 1951	IT	IT			
14. *Dermestes (Dermestinus) laniarius* Illiger, 1801					PAL
*Dermestes (Dermestinus) laniarius laniarius* Illiger, 1801	N	S	Si	Sa	
15. *Dermestes (Dermestinus) maculatus* DeGeer, 1774	N	S	Si	Sa	COS
16. *Dermestes (Dermestinus) murinus* Linnaeus, 1758					PAL
*Dermestes (Dermestinus) murinus murinus* Linnaeus, 1758	N	S	Si	Sa	
17. *Dermestes (Dermestinus) mustelinus* Erichson, 1846	N	S	Si	Sa	EUR
18. *Dermestes (Dermestinus) pardalis* Billberg, 1808	N?	IT		Sa	WME
19. *Dermestes (Dermestinus) sardous* Küster, 1846					MED
*Dermestes (Dermestinus) sardous sardous* Küster, 1846	N	S		Sa	
20. *Dermestes (Dermestinus) szekessyi* Kalík, 1950	IT	IT			SIE
21. *Dermestes (Dermestinus) undulatus* Brahm, 1790	N	S	Si	Sa	OLA
**Subgenus *Montandonia* Jacquet, 1886**					
22. *Dermestes (Montandonia) fuliginosus* P. Rossi, 1792	N	S	Si		EUR
23. *Dermestes (Montandonia) hirticollis* Fabricius, 1792				Sa	WME
24. *Dermestes (Montandonia) olivieri* Lepesme, 1939	N	S	Si	Sa	TEM
**Thorictinae Agassiz, 1846**					
**Thaumaphrastini Anderson, 1949**					
**Genus *Thorictodes* Reitter, 1875**					
25. *Thorictodes heydeni* Reitter, 1875	N	S			COS
**Thorictini Agassiz, 1846**					
**Genus *Thorictus* Germar, 1834**					
26. *Thorictus grandicollis* Germar, 1842					TUM
*Thorictus grandicollis grandicollis* Germar, 1842	N	S	Si	Sa	
27. *Thorictus mauritanicus* Lucas, 1846			Si	Sa	MED
28. *Thorictus stricticollis* Kraatz, 1859					MED
*Thorictus stricticollis stricticollis* Kraatz, 1859				Sa	
29. *Thorictus studti* John, 1971			Si		SICI
**Orphilinae LeConte, 1900**					
**Genus *Orphilus* Erichson, 1848**					
30. *Orphilus niger* (P. Rossi, 1790)	N	S	Si	Sa	CEM
**Trinodinae T.L. Casey, 1900**					
**Thylodriini Semenov, 1909**					
**Genus *Thylodrias* Motschulsky, 1839**					
31. *Thylodrias contractus* Motschulsky, 1839	N				COS
**Trinodini T.L. Casey, 1900**					
**Genus *Trinodes* Dejean, 1821**					
32. *Trinodes hirtus* (Fabricius, 1781)	N	S	Si	Sa	TUE
**Attageninae Laporte, 1840**					
**Attagenini Laporte, 1840**					
**Genus *Attagenus* Latreille, 1802**					
**Subgenus *Attagenus* Latreille, 1802**					
33. *Attagenus (Attagenus) bifasciatus* (A.G. Olivier, 1790)		S	Si	Sa	TUM
34. *Attagenus (Attagenus) brunneus* Faldermann, 1835	N	S	Si	Sa	OLA
35. *Attagenus (Attagenus) calabricus* Reitter, 1881		S	Si		APPE
36. *Attagenus (Attagenus) cyphonoides* Reitter, 1881		S			SCO
37. *Attagenus (Attagenus) fallax* Gené, 1839		S	Si	Sa	MED
38. *Attagenus (Attagenus) fasciatus* (Thunberg, 1795)	N	S			COS
39. *Attagenus (Attagenus) lobatus* Rosenhauer, 1856		S	Si	Sa	CEM
40. *Attagenus (Attagenus) maritimus* (Gené, 1839)			Si	Sa	WME
41. *Attagenus (Attagenus) obtusus* (Gyllenhal, 1808)				Sa	TEM
42. *Attagenus (Attagenus) pantherinus* (Ahrens, 1814)	N			Sa	EUR
43. *Attagenus (Attagenus) pellio* (Linnaeus, 1758)	N	S	Si	Sa	COS
44. *Attagenus (Attagenus) pilosissimus* Roubal, 1932	N	S			ALAP
45. *Attagenus (Attagenus) punctatus* (Scopoli, 1772)	N	S	Si		EUR
46. *Attagenus (Attagenus) rossii* Ganglbauer, 1904		S	Si	Sa	MED
47. *Attagenus (Attagenus) schaefferi* (Herbst, 1792)					OLA
*Attagenus (Attagenus) schaefferi schaefferi* (Herbst, 1792)	N	S	Si	Sa	
48. *Attagenus (Attagenus) silvaticus* Zhantiev, 1976		S	Si		SIE
49. *Attagenus (Attagenus) simplex* Reitter, 1881		S	Si	Sa	MED
50. *Attagenus (Attagenus) tigrinus* (Fabricius, 1792)					
*Attagenus (Attagenus) tigrinus tigrinus* (Fabricius, 1792)	N	S	Si	Sa	MED
51. *Attagenus (Attagenus) trifasciatus* (Fabricius, 1787)	N		Si?		MED
52. *Attagenus (Attagenus) unicolor* (Brahm, 1790)					COS
*Attagenus (Attagenus) unicolor unicolor* (Brahm, 1790)	N	S	Si	Sa	
53. *Attagenus (Attagenus) uniformis* Fairmaire, 1860			Si		NAF
**Megatominae Leach, 1815**					
**Anthrenini Gistel, 1848**					
**Genus *Anthrenus* Geoffroy, 1762**					
**Subgenus *Anthrenops* Reitter, 1881**					
54. *Anthrenus (Anthrenops) coloratus* Reitter, 1881		S			OLA+AFR
**Subgenus *Anthrenus* Geoffroy, 1762**					
55. *Anthrenus (Anthrenus) angustefasciatus* Ganglbauer, 1904	N	S			EUR
56. *Anthrenus (Anthrenus) delicatus* Kiesenwetter, 1851	N	S	Si	Sa	MED
57. *Anthrenus (Anthrenus) festivus* Erichson, 1846		S?	Si	Sa	MED
58. *Anthrenus (Anthrenus) flavipes* LeConte, 1854					COS
*Anthrenus (Anthrenus) flavipes flavipes* LeConte, 1854		S		Sa	
59. *Anthrenus (Anthrenus) goliath* Saulcy, 1868	N	S	Si	Sa	MED
60. *Anthrenus (Anthrenus) munroi* Hinton, 1943		S		Sa	MED
61. *Anthrenus (Anthrenus) mroczkowskii* Kalík, 1954		S			SEU
62. *Anthrenus (Anthrenus) pimpinellae* (Fabricius, 1775)					COS
*Anthrenus (Anthrenus) pimpinellae isabellinus* Küster, 1848	N	S	Si	Sa	
*Anthrenus (Anthrenus) pimpinellae pimpinellae* (Fabricius, 1775)	N	S	Si		
63. *Anthrenus (Anthrenus) scrophulariae* (Linnaeus, 1758)					COS
*Anthrenus (Anthrenus) scrophulariae scrophulariae* (Linnaeus, 1758)	N	S	Si	Sa	
**Subgenus *Florilinus* Mulsant & Rey, 1868**					
64. *Anthrenus (Florilinus) museorum* (Linnaeus, 1761)	N	S	Si	Sa	COS
65. *Anthrenus (Florilinus) oberthueri* Reitter, 1881	N		Si		WME
**Subgenus *Helocerus* Mulsant & Rey, 1868**					
66. *Anthrenus (Helocerus) fuscus* A.G. Olivier, 1790	N	S	Si	Sa	SCO
67. *Anthrenus (Helocerus) minutus* Erichson, 1846		S	Si	Sa	MED
**Subgenus *Nathrenus* Casey, 1900**					
68. *Anthrenus (Nathrenus) biskrensis* Reitter, 1887			Si		NAF
69. *Anthrenus (Nathrenus) molitor* Aubé, 1850	N			Sa	MED
70. *Anthrenus (Nathrenus) signatus* Erichson, 1846	N	S	Si		EUR
71. *Anthrenus (Nathrenus) verbasci* (Linnaeus, 1767)	N	S	Si	Sa	COS
72. *Anthrenus (Nathrenus) versicolor* Reitter, 1887			Si	Sa	MED
**Megatomini Leach, 1815**					
**Genus *Anthrenocerus* Arrow, 1915**					
73. *Anthrenocerus australis* (Hope, 1843)	N				AUST (i)
**Genus *Ctesias* Stephens, 1830**					
**Subgenus *Ctesias* Stephens, 1830**					
74. *Ctesias (Ctesias) serra* (Fabricius, 1792)	N	S	Si	Sa	EUR
**Genus *Globicornis* Latreille, 1829**					
**Subgenus *Globicornis* Latreille, 1829**					
75. *Globicornis (Globicornis) bifasciata* (Perris, 1866)			Si	Sa	WME
76. *Globicornis (Globicornis) fasciata* (Fairmaire & Brisout de Barneville, 1859)	N	S	Si?	Sa	CEU
77. *Globicornis (Globicornis) luckowi* Herrmann, Háva & Kadej, 2011	N				ALPC
78. *Globicornis (Globicornis) nigripes* (Fabricius, 1792)	N	S			EUR
79. *Globicornis (Globicornis) picta* (Küster, 1851)		S	Si	Sa	EUR
80. *Globicornis (Globicornis) tristis* (Reitter, 1881)		S			EME
81. *Globicornis (Globicornis) variegata* (Küster, 1851)	N	S	Si	Sa	SEU
**Subgenus *Hadrotoma* Erichson, 1848**					
82. *Globicornis (Hadrotoma) corticalis* (Eichhoff, 1863)	N	S	Si		EUR
83. *Globicornis (Hadrotoma) emarginata* (Gyllenhal, 1808)	N	S	Si	Sa	EUR
84. *Globicornis (Hadrotoma) sulcata* (C.N.F. Brisout de Barneville, 1866)		S	Si		WME
**Genus *Megatoma* Herbst, 1791**					
**Subgenus *Megatoma* Herbst, 1791**					
85. *Megatoma (Megatoma) ruficornis* Aubé, 1866		S			SEU
86. *Megatoma (Megatoma) undata* (Linnaeus, 1758)	N	S			EUR
**Genus *Phradonoma* Jacquelin du Val, 1859**					
87. *Phradonoma villosulum* (C. Duftschmid, 1825)	N				CAE
**Genus *Reesa* Beal, 1967**					
88. *Reesa vespulae* (Milliron, 1939)	N				SCO
**Genus *Trogoderma* Dejean, 1821**					
89. *Trogoderma angustum* Solier, 1849	IT	IT			SCO
90. *Trogoderma glabrum* (Herbst, 1783)	N	S	Si?		SCO
91. *Trogoderma granarium* Everts, 1898	N	S	Si		COS
92. *Trogoderma inclusum* LeConte, 1854	N	S	Si	Sa	COS
93. *Trogoderma megatomoides* Reitter, 1881	N				OLA
94. *Trogoderma variabile* Ballion, 1878	N			Sa	COS
95. *Trogoderma versicolor* (Creutzer, 1799)	N	S	Si	Sa	COS
**Total species**	**61**	**67**	**57**	**59**	

The following abbreviations are used in the text: dint. = dintorni = environs; env. = environs; ex = specimen/s; FA = F. Angelini leg.; FEI = Forum Entomologi Italiani (http://www.entomologiitaliani.net); Fraz. = Frazione = hamlet; GC = G. Scaglioni leg.; GNP = Gargano National Park; leg. = legit or legerunt; PC = P. Cornacchia leg.; Prog. = Progetto = Project; prov. = province; sn = sweep net.

### Acronyms of specimen depositories

AHG Andreas Herrmann private collection, Stade, Germany

CNBFVR Centro Nazionale per lo Studio e la Conservazione della Biodiversità Forestale “Bosco Fontana” di Verona, Sede di Bosco Fontana. Marmirolo (Mantua), Italy

GNAC Gianluca Nardi private collection, Cisterna di Latina (Latina), Italy

HNHM Hungarian Natural History Museum, Budapest, Hungary

JHAC Private Entomological Laboratory and Collection, Jiří Háva, Prague-west, Czech Republic

MCZR Museo Civico di Zoologia, Rome, Italy

MZUF Museo di Storia Naturale, Sezione di Zoologia, Università di Firenze, Florence, Italy

PCOP Paolo Cornacchia private collection, Porto Mantovano (Mantua), Italy

## Notes on some species

### Dermestes (Dermestes) peruvianus

Laporte de Castelnau, 1840

http://species-id.net/wiki/Dermestes_peruvianus

Dermestes peruvianus Laport.: Bertolini 1904: 58.
Dermestes peruvianus Casteln.: Luigioni 1929: 1024; Porta 1929: 301.
Dermestes (Dermestes) peruvianus Castelnau, 1840: Plata-Negrache 1972: 150; Zhantiev 2011a.
Dermestes (Dermestes) peruvianus Laporte de Castelnau, 1840: Audisio et al. 1995: 12, 16; Háva 2007: 300.
Dermestes peruvianus Laporte de Castelnau: Pollini 1998: 909.

#### Material examined.

[Latium: Roma province,] Roma env., [no other data,] 1 ex (JHAC).

#### Chorotype.

Subcosmopolitan (Háva 2007). This species is also recorded from Portugal (Grosso-Silva and Serrano 2000) and Switzerland (Kenis 2005, Zhantiev 2011a). Both countries must be added to the European distribution summarized by Háva (2007), while the former country must also be added to those provided by Zhantiev (2011a).

#### Italian distribution.

Italy? (Bertolini 1904, Luigioni 1929, Porta 1929). Italian mainland? (Audisio et al. 1995). Italy (without further details) (Plata-Negrache 1972, Háva 2007). Italian mainland (Zhantiev 2011a).

#### Remarks.

Species described from Peru (cf. Háva and Kalík 2005); the first precise record from Italy is shown above.

Domenichini et al. (1993: 272) wrote that this species is *common in food factories* and was reared experimentally in an insectarium in Piacenza University (northern Italy), but the place of origin of this captive population is not mentioned. This species, and *Dermestes (Dermestinus) carnivorus* Fabricius, 1775 (see below), are also considered in an Italian manual of applied entomology (Pollini 1998), but no records from Italy are listed therein.

### Dermestes (Dermestinus) carnivorus

Fabricius, 1775

http://species-id.net/wiki/Dermestes_carnivorus

Dermestes carnivorus Fabricius: Pollini 1998: 906.
Dermestes (Dermestinus) carnivorus Fabricius, 1775: Háva 2007: 300; Zhantiev 2011a.

#### Material examined.

Tuscany: Firenze [= Florence], V.1948, [no collector,] 1 ♀ (JHAC).

#### Chorotype.

Cosmopolitan (cf. Háva 2007).

#### Italian distribution.

Italy (without further details) (Háva 2007). Italian mainland (Zhantiev 2011a).

#### Remarks.

Pollini (1998: 906) wrote about this species that *si è acclimatato in Europa e in India[= it is naturalized in Europe and India]*, but no Italian locality was mentioned. The above generic Italian record by Háva (2007), was based on the above specimen.

### Dermestes (Dermestes) vorax

Motschulsky, 1860

http://species-id.net/wiki/Dermestes_vorax

Dermestes vorax Motschulsky, 1860: Denux and Zagatti 2010: 357.
Dermestes (Dermestes) vorax Motschulsky, 1860: Háva and Nardi 2011: 408.

#### Material examined.

None.

#### Chorotype.

Asiatic (Háva 2003, 2007). This species was introduced to the coastal Croatia (Depoli 1928, Luigioni 1929, Porta 1934, all as *Dermestes vorax* Motsch.), but this record was later ignored (Mroczkowski 1968, Háva 2003, 2007, Zhantiev 2011a).

#### Italian distribution.

Italy (without further details) (Denux and Zagatti 2010).

#### Remarks.

Denux and Zagatti (2010) wrote that an Italian record of this species was provided by Lohse (1979) and Háva (2003), but in these papers this species is not recorded from this country. Moreover, a recent record from “Italy” (http://www.dermestidae.wz.cz/main.html; http://www.dermestidae.com/index.html) is erroneous since concerns a dark specimen of *Dermestes (Dermestes) lardarius* Linnaeus, 1758 (Hava, unpublished data), so it was ignored by Háva and Nardi (2011).

### Dermestes (Dermestinus) hankae

Háva, 1999

http://species-id.net/wiki/Dermestes_hankae

Dermestes (Dermestinus) hankae Háva, 1999: Háva 2007: 301.
Dermestes (Dermestinus) hankai [sic!] Háva, 1999: Zhantiev 2011a.

#### Material examined.

Italy mer. [= southern Italy], [no other data], 1 ♂ (JHAC).

#### Chorotype.

W-Mediterranean: Spain, France, Italy and Algeria (Háva 2007, Prieto and Háva 2013).

#### Italian distribution.

Italy (without further details) (Háva 2007). Italian mainland (Zhantiev 2011a).

#### Remarks.

Species described from southern France (Háva 1999: 143); the generic record for Italy (Háva 2007) was based on the above specimen.

The specific name (*hankae*) is correct (ICZN 1999, art. 31.1.2), so the name *hankai* is only a subsequent incorrect spelling.

This species is very similar to *Dermestes (Dermestinus) pardalis* Billberg, 1808 (Háva 1999, Herrmann and Bahillo de la Puebla 2003, Prieto and Háva 2013); the re-examination of specimens attributed to it could provide additional records of *Dermestes (Dermestinus) hankae*. In Italy, *Dermestes (Dermestinus) pardalis* is recorded from Sardinia (cf. Háva and Nardi 2011), generically from mainland Italy (Zhantiev 2011a), and from a non-specified locality of the northern mainland (Giachino 1982: 360, as *Dermestes thoracicus* Dejean[, 1821 (nomen nudum)]), but this record, based on very old specimens, should be confirmed.

### Dermestes (Dermestinus) intermedius intermedius

Kalík, 1951

http://species-id.net/wiki/Dermestes_intermedius_intermedius

Dermestes (Dermestinus) intermedius intermedius Kalík, 1951: Háva 2003: 18; Háva 2007: 301.
Dermestes (Dermestinus) intermedius Kalík, 1951: Zhantiev 2011a.

#### Material examined.

None.

#### Chorotype.

Turano-European reaching only as far as (*Dermestes (Dermestinus) intermedius iranicus* Háva & Kalík, 1999) Iran, Iraq and Syria eastward (cf. Háva 2007, Háva et al. 2011).

#### Italian distribution.

Italy (without further details) (Háva 2003, 2007). Italian mainland (Zhantiev 2011a).

#### Remarks.

The above generic Italian records by Háva (2003, 2007) were based on a personal communication of V. Kalík, who identified an Italian specimen; unfortunately, no further details are available (Háva, unpublished data).

### Dermestes (Dermestinus) szekessyi

Kalík, 1950

http://species-id.net/wiki/Dermestes_szekessyi

Dermestes (Dermestinus) szekessyi Kalík, 1950: Háva 2007: 302; Zhantiev 2011a.

#### Material examined.

None.

#### Chorotype.

Sibero-European (cf. Háva 2003, 2007, Háva et al. 2011, Zhantiev 2011a).

#### Italian distribution.

Italy (without further details) (Háva 2007). Italian mainland (Zhantiev 2011a).

#### Remarks.

The above generic Italian record of Háva (2007) was based on a personal communication of V. Kalík, who identified an Italian specimen; unfortunately, no further details are available (Háva, unpublished data).

### Dermestes (Montandonia) olivieri

Lepesme, 1939

http://species-id.net/wiki/Dermestes_olivieri

Dermestes ater Oliv. [= Olivier, 1790]: Ghiliani 1842: 33; Ghiliani 1847: 93; Reiche 1860: 720; Ghiliani 1887: 310; Bertolini 1890: 196; Iehl 1909: 26; Luigioni 1929: 537; Porta 1929: 301; Gridelli 1949: 176; Horion 1955: 197; Faggioli 1956: 174; Gulli 1961: 4.
Dermestes ater Ol.: Villa and Villa 1844: 38 [444]; Rottenberg 1870: 238; Steck 1887: 181; Ragusa 1892: 203; Bertolini 1904: 58; Vitale 1904: 75; Holdhaus 1911: 445; Panganetti-Hummler 1918: 87; Zangheri 1969: 1334.
Dermestes ater Olivier: Giachino 1982: 361.
Dermestes olivieri Lep.: Angelini 1986: 74; Angelini and Montemurro 1986: 573; Angelini 1987: 38; Angelini 1991: 206.
Dermestes (Dermestes) olivieri Lepesme, 1939: Audisio et al. 1995: 12; Bordoni et al. 2006: 60; FEI 2011.
Dermestes ater Degeer, 1774 [sic!]: Sapuppo 2002: 189.
Dermestes (Montandonia) olivieri Lepesme, 1939: Háva and Nardi 2011: 416.

#### Material examined.

Apulia: Foggia prov., [GNP,] Mattinata, strada per M.te Sacro [= road to Mount Sacro], Masseria Vaira dint., 613 m, 33T 584160 4821825, 24.V.2010, I. Toni leg., sn on *Ferula communis* [(Apiaceae)], Prog. Foresta Umbra, 1 ex (CNBFVR); Taranto prov., Ginosa Marina, VIII.1985, P. Abbazzi leg., 2 ex (MZUF). Tuscany: Arezzo [prov.], Cavriglia, [Fraz.] Montegonzi, 16.V.1991, Lisa leg., 1 ex (MZUF); [Arezzo prov.,] Ris. Nat. [= Riserva Naturale = Nature Reserve] “Valle Inferno-Bandella”, Casa Giardino, 8.V.1998, L. Bartolozzi, B. Cecchi A. & Sforzi leg., 1 ex (MZUF); [Firenze prov.,] Firenze [= Florence], V.1896, ex-coll. Beccari, 1 ex (MZUF); Firenze prov., Greve [in Chianti], [Fraz.] Strada in Chianti, 20.I.1957, A. Bandinelli leg., 1 ex (MZUF); [Grosseto prov.], Parco Naturale della [= Natural Park of] Maremma, Mti [= Monti = Mounts] dell’Uccellina, S. Rabano, X.1988, L. Bartolozzi leg., 4 ex (MZUF).

#### Chorotype.

Turano-European with extension to Tunisia (cf. Háva 2007, Háva and Herrmann 2009, 2011, Háva et al. 2011, Háva and Nardi 2011).

#### Italian distribution.

All of Italy, Sicily and Sardinia (cf. Bertolini 1904, Luigioni 1929, Horion 1955, Audisio et al. 1995, Háva and Nardi 2011). These generic distributional data are most likely correct; nevertheless, precise records are known only from the following regions: Trentino (Bertolini 1890), Lombardy (Villa and Villa 1844, Giachino 1982), Piedmont (Ghiliani 1847, 1887), Aosta Valley (Iehl 1909), Romagna (Zangheri 1969), Tuscany (Bordoni et al. 2006), Latium (Horion 1955, FEI 2011), Abruzzi (Horion 1955), Apulia (Holdhaus 1911, Panganetti-Hummler 1918, Gridelli 1949, Horion 1955, Faggioli 1956, Angelini 1987), Basilicata (Angelini 1986, Angelini and Montemurro 1986), Calabria (Angelini 1991), Sicily (Ghiliani 1842, Rottenberg 1870, Steck 1887, Ragusa 1892, Vitale 1904, Luigioni 1929, Horion 1955, Audisio et al. 1995), and Sardinia (cf. Háva and Nardi 2011).

#### Remarks.

New collecting sites from Tuscany and Apulia. In Tuscany, the species was formally recorded from a sole locality (Bordoni et al. 2006); it is also for the first time recorded here from a nature reserve, of which the beetle-fauna is well known (cf. Zinetti and Terzani 2009). The species was already known from Apulia, from various localities, including the Gargano National Park (see the references above).

Sapuppo (2002) recorded *Dermestes ater* DeGeer, 1774 from two Sicilian localities based on specimens collected by A. Gulli and identified by A. Porta. The data from a locality are exactly the same as those of “*Dermestes ater* Oliv.” provided by Gulli (1961), and thus, both sites are attributed here to *Dermestes (Montandonia) olivieri*; the homonymy (*Dermestes ater* DeGeer and *Dermestes ater* Oliver) was very probably the cause of the error of Sapuppo (2002). *Dermestes (Dermestes) ater* DeGeer is a cosmopolitan species, but in Italy it is known from some northern regions only (Luigioni 1929: 537, as *Dermestes (Dermestes) cadaverinus* Fabr. [= Fabricius, 1775], Minelli and Negrisolo 1993: 92, as *Dermestes ater* Degeer, Audisio et al. 1995: 12, as *Dermestes (Dermestes) ater* Degeer).

### Thorictus pilosus

Peyron, 1857

http://species-id.net/wiki/Thorictus_pilosus

Thorictus pilosus Peyron, 1857: Audisio et al. 1995: 12.

#### Material examined.

None.

#### Chorotype.

Turanian-E-Mediterranean: Cyprus, Egypt, Greece, Iraq, Israel, Lebanon, Libya, Syria, Tunisia, Turkey and Uzbekistan (John 1963, Löbl 2007).

#### Italian distribution.

“S?” [= peninsular Italy?] (Audisio et al. 1995).

#### Remarks.

The above doubtful record for peninsular Italy has already been ignored by other authors (Löbl 2007, Zhantiev 2011a); this species is excluded from Italy here, since when considering its geographical distribution, its occurrence in this country is improbable. A doubtful record from Corsica (cf. Bertolini 1874: 96, as *Thorictus piliger* Schaum[, 1858], Luigioni 1929: 1022, as *Thorictus pilosus* Peyr., Porta 1929: 204, as *Thorictus pilosus* Peyron) has also never been confirmed (Löbl 2007, Zhantiev 2011a), and must be referred to *Thorictus grandicollis grandicollis* Germar, 1842 (Háva, unpublished data).

### Thorictus wasmanni

Reitter, 1895

http://species-id.net/wiki/Thorictus_wasmanni

Thorictus wasmanni Reitter, 1895: Audisio et al. 1995: 12.

#### Material examined.

None.

#### Chorotype.

Turanian: endemic of Uzbekistan (John 1963, Löbl 2007).

#### Italian distribution.

“Si?” [= Sicily?] (Audisio et al. 1995).

#### Remarks.

The above doubtful Sicilian record has already been ignored by other authors (Löbl 2007, Zhantiev 2011a); this species must be excluded from the Italian fauna, since its occurrence in Sicily is incompatible with its geonemy. It is known from the type specimens only (Zhantiev 2011b).

### Attagenus (Attagenus) calabricus

Reitter, 1881

http://species-id.net/wiki/Attagenus_calabricus

Attagenus calabricus Reitter, 1881: 37.
Attagenus (Lanorus) calabricus Reitt.: Reitter 1883: 89; Reitter 1891: 337; Ragusa 1892: 204; Reitter 1906: 378; Dalla Torre 1911: 52; Winkler 1926: 676; Luigioni 1929: 538; Porta 1929: 302.
Megatoma calabrica Rttr.: Reitter 1887: 48.
Attagenus calabricus Reitt.: Bertolini 1904: 58; Panganetti-Hummler 1918: 87; Schatzmayr 1941: 73; Angelini 1986: 74; Angelini and Montemurro 1986: 573; Angelini 1987: 38; Angelini 1991: 206.
Attagenus calabricus Rtt.: Liebmann 1962: 4.
Attagenus calabricus Reitter, 1881: Mroczkowski 1968: 82; Sparacio 1997: 73.
Attagenus (Lanorus) calabricus Reitter, 1881: Audisio et al. 1995: 13.
Attagenus (Attagenus) calabricus Reitter, 1881: Háva 2003: 59; Háva 2007: 308; Zhantiev 2011a.

#### Material examined.

Abruzzi: Pescara prov., Maiella, Valle Orfento, Caramanico, 23.VI.1988, G. Osella leg., 1 ex (JHAC). Apulia: Brindisi prov., Francavilla Fontana, 30.V.1998, C. Esposito leg., 1 ex (GNAC); [Foggia prov., GNP,] Mattinata, 22.VI.1991, on flowers on the beach, M. Mantic leg., 4 ex (JHAC); [Foggia prov., GNP,] Gargano, Mattinata, 8–10.V.1995, Z. Svec leg., 1 ex (JHAC); [Foggia prov., GNP, Vico del Gargano, Fraz.] San Menaio, V.1996, M. Fikacek leg., 2 ex (JHAC); Taranto prov., Circ. [= Circum] Mar Piccolo, 24.V.1992, Montemurro leg., 1 ex (JHAC); Taranto prov., Grottaglie, VI.1369 [sic!], FA, 1 ex (MCSV). Basilicata: [Matera prov.,] Marina di Nova Siri, 4.VI.2008, W. Apfel leg., 1 ex (JHAC); [Matera prov.,] Matera, 4.VI.1989, FA, 1 ex (AHG); [Matera prov.,] Matera, 350 m, 4.VI.1989, FA, 11 ex (MCSV); Potenza prov., Francavilla sul Sinni, 320 m, 5.VI.1988, querceta [= oak wood], FA, 1 ex (MCSV); [Potenza or Matera prov.,] Pollino, 19.VI.1994, FA, 1 ex (AHG). Calabria: “Calabria / Baudi [leg.]” [handwritten by unknown], “Holotypus 1881 / Attagenus / Calabricus / Reitter” [red-framed; handwritten by unknown], “Calabricus m. / Calab. Baudi [leg.]” [handwritten probably by Reitter], “coll. Reitter” [printed], “Lectotype / *Attagenus calabricus* Reitter / J. Háva des. 2012”, 1 ex (HNHM); “Calabria / Baudi [leg.]” [handwritten by unknown], “Paratypus 1881 / Attagenus / Calabricus / Reitter” [red-framed; handwritten by unknown], “coll. Reitter [printed]”, “Paralectotype / *Attagenus calabricus* Reitter / J. Háva des. 2012”, 1 ex (HNHM); Calabria [without further data], 1 ex (JHAC); [Cosenza prov., Cassano all‘Ionio, Fraz.] Marina di Sibari env., 3 m, sandy dunes, 14.6.2009, P. Kresl leg., 6 ex (JHAC); [Cosenza prov.,] Roseto Capo Spulico, 27.V.–3.VI.2006, V. Hron leg., 1 ex (JHAC); [Cosenza prov.,] Sila, Mte. [= Mount] Oliveto, 2.VII.1929, C. Confalonieri leg., 1 ex (JHAC); [Cosenza prov.,] Sila, Sud [= South of] S. Giovanni [in] Fiore, 1100 m, 4.VII.[19]87, FA, 2 ex (MCSV); Reggio Calabria prov., Asprom. [= Aspromonte], str. [= strada = road] Cimina–Zomaro, 600–800 m, 6.VI.1994, FA, 6 ex (JHAC).

#### Chorotype.

Apenninic endemic (Háva 2007).

#### Italian distribution.

Campania (Luigioni 1929), Apulia (Panganetti-Hummler 1918, Luigioni 1929, Angelini 1987), Basilicata (Angelini 1986, Angelini and Montemurro 1986), Calabria (Bertolini 1904, Luigioni 1929, Porta 1929, Schatzmayr 1941, Angelini 1991) and Sicily (Ragusa 1892, Bertolini 1904, Winkler 1926, Luigioni 1929, Porta 1929, Liebmann 1962, Mroczkowski 1968, Audisio et al. 1995, Sparacio 1997, Háva 2003, 2007, Zhantiev 2011a).

The generic records for Italy (Reitter 1883, 1891, 1906, Dalla Torre 1911, Winkler 1926, Mroczkowski 1968, Háva 2003, 2007), peninsular Italy (Audisio et al. 1995), southern Italy (Sparacio 1997) and mainland Italy (Zhantiev 2011a) refer to the above listed regions.

#### Remarks.

First record from the Abruzzi region; this new site (cf. Osella 1988) is at the northernmost limit of the species distribution, while the above record from the Brindisi province is at the easternmost limit. The southernmost site is the Pantelleria Island in the Sicilian Channel (Liebmann 1962).

Reitter (1881) described this species without giving any locality of collecting (see also Reitter 1887), but based on the specific name, the type locality is apparently in the Calabria region (southern peninsular Italy). The species was described based on an unspecified number of specimens without any type designation (Reitter 1881). The two type specimens above are thus syntypes and are designated here as lectotype and paralectotype, respectively; in this way “Calabria” becomes the type locality of the species (ICZN 1999, art. 76.2).

### Attagenus (Attagenus) lobatus

Rosenhauer, 1856

http://species-id.net/wiki/Attagenus_lobatus

Attagenus (Attagenus) lobatus Rosenhauer, 1856: Háva and Nardi 2011: 420.

#### Material examined.

Tuscany: Firenze [= Florence], 28.IX.1988, L. Chelazzi leg., 1 ♂ (MZUF).

#### Chorotype.

Centralasiatic-Mediterranean; this species was introduced to the USA (cf. Háva and Nardi 2011; Háva et al. 2013).

#### Italian distribution.

Latium, Campania, Apulia, Calabria, Sicily and Sardinia (cf. Háva and Nardi 2011).

#### Remarks.

First record for Tuscany. The new locality is the northernmost in Italy (cf. Háva and Nardi 2011).

### Attagenus (Attagenus) pilosissimus

Roubal, 1932 stat. prom.

Attagenus pellio L. var. *pilosissimus* Roubal, 1932: 66.
Attagenus pellio Lin. v. *pilosissimus* Roubal: Porta 1934: 171.
Attagenus pellio (Linnaeus, 1758) var. *pilosissimus* Roubal, 1932: Mroczkowski 1968: 89.
Attagenus pellio var. *pilosissimus* Roub.: Angelini 1986: 74.
Attagenus pellio var. *pilosissimus* Roubal: Angelini 1991: 206.
Attagenus (Attagenus) pellio (Linné, 1758) = *Attagenus pilosissimus* Roubal, 1932: Audisio et al. 1995: 13.
Attagenus (Attagenus) pellio (Linnaeus, 1758) = *Attagenus pellio* var. *pilosissimus* Roubal, 1932: Háva 2003: 66.
Attagenus (Attagenus) pellio (Linnaeus, 1758) = *Attagenus pilosissimus* Roubal, 1932: Háva 2007: 57.
Attagenus (Attagenus) pellio (Linnaeus, 1758) = *Attagenus pellio* var. *pilosissimus* Roubal, 1932: Háva and Nardi 2007: 122.

#### Material examined.

Apulia: Bari prov., Gioia del Colle, 22.IV.1993, De Marzo leg., 1 ♂ (JHAC); Foggia prov., [GNP,] Foresta Umbra, Monte Sant’Angelo, caserme forestali [= barracks of the Italian National Forestry Service], 792 m, 33T 582306 4630267, 27.IV.2010, PC, death on windowsill, Prog. Foresta Umbra, 1 ♀ (JHAC). Lombardy: Bergamo prov., Oltre il Colle, 7.VII.2005, FA, 1 ♂ (JHAC).

#### Chorotype.

Alpino-Apenninic endemic (cf. Háva and Nardi 2007).

#### Italian distribution.

Apulia and Calabria (Roubal 1932, Porta 1934, Angelini 1986, 1991, Háva and Nardi 2007).

#### Remarks.

**Figures 1–6.**
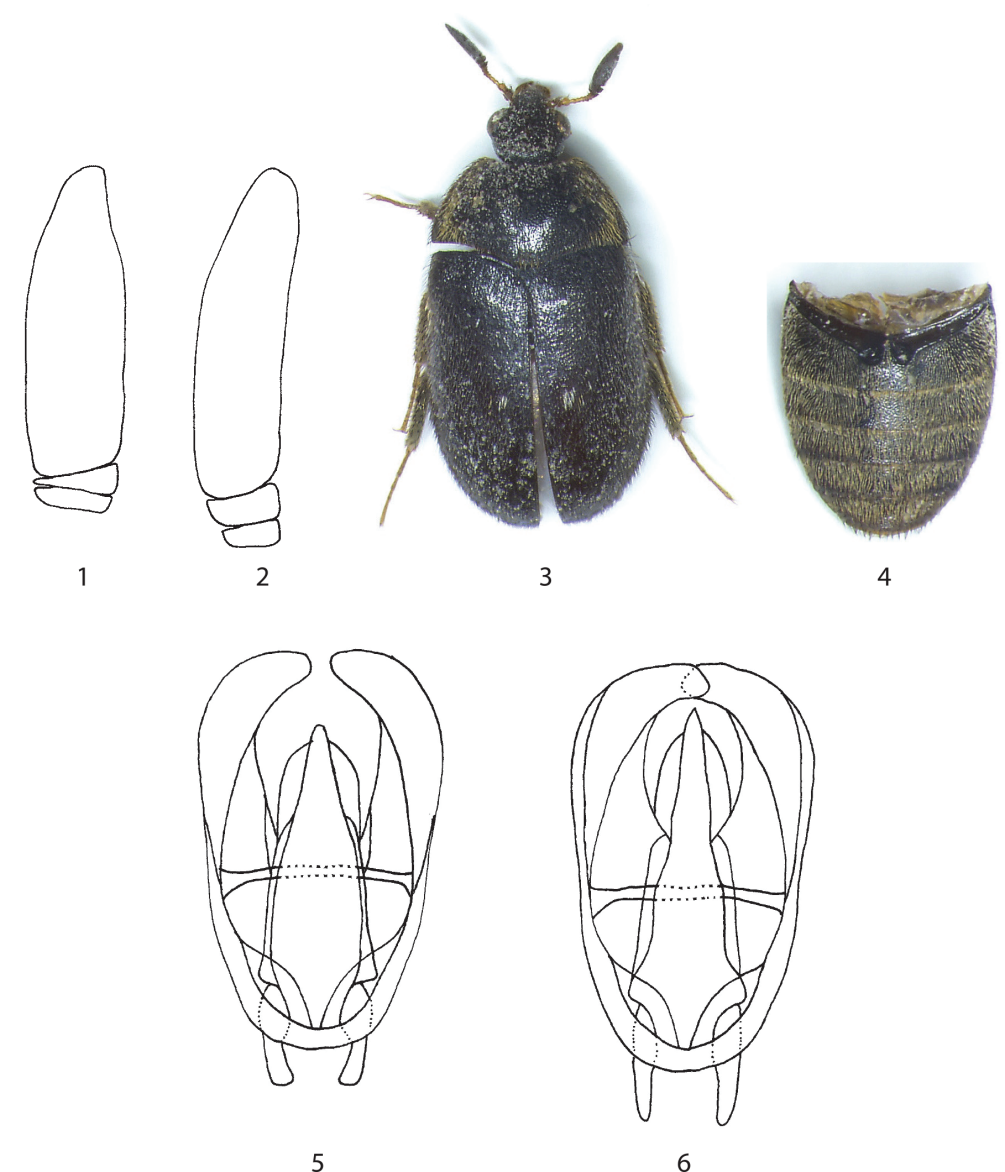
*Attagenus (Attagenus)* spp., males **1** Antennal club of *Attagenus (Attagenus) pilosissimus* from Italy (Apulia, Bari prov., Gioa del Colle) **2** Antennal club of *Attagenus (Attagenus) pellio* from Czech Republic (Central Bohemia, Prague) **3** Habitus of *Attagenus (Attagenus) pilosissimus* from Italy (Apulia, Bari prov., Gioia del Colle) (length 4.5 mm) **4** Abdomen in ventral view of *Attagenus (Attagenus) pilosissimus* from Italy (Apulia, Bari prov., Gioa del Colle) **5** Aedeagus (setation omitted) of *Attagenus (Attagenus) pilosissimus* from Italy (Lombardy, Bergamo prov., Oltre il Colle) (length 0.5 mm) **6** Aedeagus (setation omitted) of *Attagenus (Attagenus) pellio* from Czech Republic (Central Bohemia, Prague) (length 0.5 mm) (photos and drawings by J. Háva).

*Attagenus pellio* var. *pilosissimus* from Apulia was listed as a synonym of *Attagenus (Attagenus) pellio* by Audisio et al. (1995), because Mroczkowski (1968) listed *pilosissimus* as a “variety”. This means that this name was implicitly considered as a junior synonym as Mroczkowski also treated subspecific taxa in his paper. This opinion was confirmed by Háva and Nardi (2007: 122) through a study of the holotype, a female specimen which is very worn. A photo of this holotype is published at http://www.dermestidae.com/Attagenuspelliopilosissimus.html. Nevertheless, based on the study of the above new material, this variety is resurrected as a valid species (stat. prom.). *Attagenus (Attagenus) pilosissimus* and *Attagenus (Attagenus) pellio* show differences in the shape of the antennal club (Figs 1–2) and in the male genitalia (Figs 4–5): the median lobe of the aedeagus of the former species (Fig. 5) is longer and narrower, and parameres are parallel for most of their length. Moreover, *Attagenus (Attagenus) pilosissimus* also differs by the pubescence of the abdomen (Fig. 4) and pronotum (Fig. 3) and by the presence of only one small circular spot with whitish pubescence on each elytron (Fig. 3). *Attagenus (Attagenus) pilosissimus* is known only from mainland Italy (Lombardy, Apulia and Calabria), where, in sympatry, *Attagenus (Attagenus) pellio* (Linnaeus, 1758) also occurs (cf. Holdhaus 1911, as *Attagenus pellio* L., Háva and Nardi 2007).

### Attagenus (Attagenus) silvaticus

Zhantiev, 1976

http://species-id.net/wiki/Attagenus_silvaticus

Attagenus (Attagenus) silvaticus Zhantiev, 1976: Háva and Nardi 2007: 122.
Attagenus silvaticus Zhantiev, 1976: Zhantiev 2011a.

#### Material examined.

Apulia: Foggia prov., [GNP,] Foresta Umbra, Vico del Gargano, Bosco Sfilzi, 391 m, 33T 585633 4634653, 23.V.2010, D. Birtele & I. Toni leg., window flight trap, Prog. Foresta Umbra, 1 ♀ (CNBFVR).

#### Chorotype.

European (Czech Republic, Hungary, northwestern Russia, Sicily, Slovakia, Ukraine) with extension eastward to “Caucasus”, Anatolia, Iran and West Siberia (Háva 2007, Háva and Nardi 2007, Háva et al. 2010, as *Attagenus silvaticus*, Zhantiev 2011a).

#### Italian distribution.

Sicily (Háva and Nardi 2007, Zhantiev 2011a).

#### Remarks.

First record for mainland Italy. The above specimen was intercepted in a beech wood (D. Birtele, pers. comm., 2012).

### Attagenus (Attagenus) simonis

Reitter, 1881

http://species-id.net/wiki/Attagenus_simonis

Attagenus tigrinus F. [= (Fabricius, 1792)] v. *Simoni* [sic!] Reit.: Bertolini 1904: 58.
Attagenus bifasciatus Oliv. [= (Olivier, 1790)] v. *Simoni* [sic!] Reitt.: Luigioni 1923b: 4.
Attagenus (Lanorus) bifasciatus Oliv. a *Simoni* [sic!] Reitt.: Luigioni 1929: 538; Porta 1929: 302.
Attagenus bifasciatus (Olivier, 1790) var. *simoni* [sic!] Reitter, 1881: Sapuppo 2002: 187–188.
Attagenus (Attagenus) simonis Reitter, 1881: Háva and Nardi 2007: 418–419.

#### Material examined.

None.

#### Chorotype.

E-Mediterranean: Egypt, Israel, Jordan and Syria (Háva 2007).

#### Italian distribution.

Latium (Luigioni 1929), Campania (Bertolini 1904, Luigioni 1929, Porta 1929), Capri Island (Luigioni 1923b, 1929, Porta 1929) and Sicily (Luigioni 1929, Sapuppo 2002).

#### Remarks.

This species was recently excluded from the Italian fauna (Háva and Nardi 2007, 2011); also according to Mroczkowski (1968: 81, as *Attagenus bifasciatus* var. *simonis*), it occurs only in some East-Mediterranean countries. Nevertheless, an escatomediterranean distributional pattern (Zilli 2000) could explain the occurrence of this species in Italy.

Gulli (1961: 4, as *Attagenus bifasciatus* Oliv. var. *Rossii* Ganglb. [= Ganglbauer, 1904] and *Attagenus bifasciatus* var. *Simoni* [sic!]) recorded Sicilian specimens of *Attagenus (Attagenus) rossii* (identified by A. Porta) with a colour similar to *Attagenus (Attagenus) simonis*; these specimens are also listed by Sapuppo (2002), but unfortunately their re-examination has so far been impossible.

### Attagenus (Attagenus) tigrinus pulcher

Faldermann, 1835

http://species-id.net/wiki/Attagenus_tigrinus_pulcher

Attagenus pulcher Fald.: Bargagli 1872: 102, Bertolini 1874: 99.
Attagenus tigrinus F. [= (Fabricius, 1792)] var. *pulcher* Falder.: Bertolini 1904: 58.

#### Material examined.

None.

#### Chorotype.

Mediterranean (cf. Háva and Nardi 2011); *Attagenus (Attagenus) tigrinus pulcher* is a Caucasian endemic (Zhantiev 2009).

#### Italian distribution.

Sardinia (Bargagli 1872, Bertolini 1874, 1904).

#### Remarks.

This subspecies is excluded here from the Italian fauna. Zhantiev (2009) recognized this taxon as a valid subspecies. This paper was overlooked by Háva and Nardi (2011), so these authors, according to Háva (2007), listed it as a synonym of *Attagenus (Attagenus) bifasciatus* (A.G. Olivier, 1790). Nevertheless, the status of this subspecies needs to be revised (Háva, unpublished data).

### Anthrenus (Anthrenops) coloratus

Reitter, 1881

http://species-id.net/wiki/Anthrenus_coloratus

Anthrenus (Anthrenops) coloratus Reitter, 1881: Háva 2007: 311.
Anthrenus (Florilinus) coloratus Reitter, 1881: Zhantiev 2011a.

#### Material examined.

Apulia: [Foggia prov., GNP,] Gargano, V.1991, J. Háva leg., 12 ex (JHAC).

#### Chorotype.

Turano-Europeo-Mediterranean with extension to Canary Islands, Tajikistan, “India” and Sudan; this species was introduced to and is widespread in the USA (Beal 1998, as *Anthrenus coloratus*, Háva 2003, 2007).

#### Italian distribution.

Italy (Háva 2007). Italian mainland (Zhantiev 2011a).

#### Remarks.

The generic Italian record by Háva (2007) was based on the above specimens.

### Anthrenus (Anthrenus) angustefasciatus

Ganglbauer, 1904

http://species-id.net/wiki/Anthrenus_angustefasciatus

Anthrenus (Anthrenus) pimpinellae Fabr. [= (Fabricius, 1775)] a. *angustefasciatus* Ganglb.: Luigioni 1929: 540.
Anthrenus pimpinellae Fabr. ab. *angustefasciatus* Ganglb.: Porta 1934: 171.
Anthrenus (Anthrenus) angustefasciatus Ganglbauer, 1904: Háva 2003: 79; Háva 2007: 311; Kadej et al. 2007: 728; Háva 2011b: 78; Zhantiev 2011a; Háva et al. 2013: 137.

#### Material examined.

Friuli-Venezia Giulia: Trieste prov., Carso, [Sgonico, Fraz.] Gabrovizza S. Primo, 12.VI.1988, sn, PC, 1 ♀ (PCOP). Tuscany: Grosseto prov., Pitigliano, 23.I.[20]02, L. Saltini leg., 1 ex (GNAC). Apulia: [Brindisi prov.,] Brindisi env., 18.V.1997, J. Hrdlicka leg., 4 ex (JHAC). Basilicata: Potenza prov., [Pollino National Park,] Terranova di Pollino, 900 m, 27–30.VI.2005, GC, sn, 1 ♀ (PCOP).

#### Chorotype.

European with extension southward to a part of the Maghreb: Algeria, Bosnia and Herzegovina, Croatia, Czech Republic, France mainland, Italy, Morocco, Portugal, Spain, Switzerland, Yugoslavia and European Turkey (cf. Háva 2011b, Zhantiev 2011a, Háva et al. 2013).

The records for Corsica (Luigioni 1929, Porta 1934) were later ignored (Sainte-Claire Deville 1937, Mroczkowski 1968, as *Anthrenus (Anthrenus) pimpinellae* var. *angustefasciatus*, Zhantiev 2011a, Háva et al. 2013).

#### Italian distribution.

Calabria (Luigioni 1929, Porta 1934), Sardinia (Háva et al. 2013). Italy (without further details) (Háva 2003, 2007, Kadej et al. 2007, Háva 2011b). Italian mainland (Zhantiev 2011a).

#### Remarks.

Ganglbauer (1904: 42) described *Anthrenus pimpinellae* var. *angustefasciatus* from “Dalmatien [= Dalmatia]” that was only recently raised to species level (Háva 2003: 79); its diagnostic features were provided by different authors (Kadej 2005, Kadej et al. 2007, Háva 2011b, Háva and Zahradník 2011).

First records for northern Italy (Friuli-Venezia Giulia), for central Italy (Tuscany), for Apulia and Basilicata; the former record is expected since this species occurs in neighbouring foreign regions (Depoli 1928, as *Anthrenus pimpinellae* f. *angustefasciatus* Ganglb., Novak 1952, as *Anthrenus pimpinellae* a. *angustefasciatus* Gglb.). The generic records for Italy (Háva 2003, 2007, Kadej et al. 2007, Háva 2011b) and, probably also those generic for mainland Italy (Zhantiev 2011a), refer to previous literature records and, in part, to the above material.

### Anthrenus (Anthrenus) delicatus

Kiesenwetter, 1851

http://species-id.net/wiki/Anthrenus_delicatus

Anthrenus pimpinellae Fabr. [= (Fabricius, 1775)] var. *delicatulus* [sic!] Kiesw.: Ghiliani 1887: 311.
Anthrenus pimpinellae F. var. *delicatus* Kiesw.: Steck 1887: 181; Ragusa 1892: 206.
Anthrenus pimpinellae Fabr. Var. *delicatulus* [sic!] Kiesw.: Baudi 1890: 102.
Anthrenus pimpinellae F. [var.] *Isabellinae* Muls. [= Mulsant & Rey, 1868]: Ragusa 1892: 206.
Anthrenus pimpinellae F. var. *delicatus* K.: Bertolini 1904: 59.
Anthrenus pimpinellae F. ab. *delicatus* Kiesw.: Luigioni 1923a: 135.
Anthrenus (Anthrenus) pimpinellae F. a. *delicatus* Kiesw.: = *Isabellinae* Muls. Rey: Luigioni 1929: 540.
Anthrenus (Anthrenus) pimpinellae Fabr. V. *delicatus* Kiesw.: Porta 1929: 306.
Anthrenus pimpinellae v. *delicatus* Kiesw.: Liebmann 1962: 4.
Anthrenus (Anthrenus) delicatus Kiesenwetter, 1851: Audisio et al. 1995: 14; Kadej 2005: 728; Lo Cascio et al. 2006: 322; Háva and Nardi 2011: 423; Zhantiev 2011a.
Anthrenus (Anthrenus) delicatus delicatus Kiesenwetter, 1852 [sic!]: Háva 2007: 312.

#### Material examined.

Apulia: Foggia prov., [GNP,] Promontorio del Gargano, Vieste, SP 89 [= Strada Provinciale n. 89 = Provincial Road n. 89], Casa Cupari dint., 354 m, 33T 5920001 4630150, 29.V.2010, abandoned quarry, sn on grasses, PC, Prog. Foresta Umbra, 6 ex (CNBFVR; JHAC); Foggia prov., [GNP,] Promontorio del Gargano, Peschici, Lampia del Principe dint., 161 m, 33T 587282 4639346, 28.IV.2010, maquis sn, PC, Prog. Foresta Umbra, 1 ♀ (CNBFVR). Latium: Roma prov., M.ti [= Monti = Mounts] Simbruini, Jenne dint., M.te [= Monte = Mount] Porcaro, 800 m, 31.V.2007, sn, PC GC, 1 ex (PCOP). Sicily: Palermo prov., Corleone, Bosco della Ficuzza, 700 m, 1.VI.2008, sn near a watering trough, PC GC, 1 ex (PCOP).

#### Chorotype.

Mediterranean, reaching eastward as far as Caucasus and Iran (cf. Kadej 2005, Háva and Nardi 2011, Zhantiev 2011b). This species is also recorded from Cyprus (Zhantiev 2011a, Alziar and Lemaire 2012: 67, as *Anthrenus delicatus*), and this country must be added to the distribution summarized by Háva and Nardi (2011).

#### Italian distribution.

Trentino, Venezia Giulia (Luigioni 1929), Piedmont (Ghiliani 1887, Baudi 1890, Bertolini 1904, Luigioni 1929), Lombardy (Háva and Nardi 2004), Liguria (Luigioni 1929), Latium, Campania (Luigioni 1923a, Porta 1929), Sicily (Ragusa 1892, Bertolini 1904, Luigioni 1929, Porta 1929, Liebmann 1962, Audisio et al. 1995, Lo Cascio et al. 2006) and Sardinia (Bertolini 1904, Luigioni 1929, Porta 1929, Audisio et al. 1995, Háva and Nardi 2011, Zhantiev 2011a).

Moreover, Luigioni (1929) recorded this species generically for central and southern Italy; generic records for northern and peninsular Italy (Audisio et al. 1995) and for “Italy” (Kadej 2005, Háva 2007) refer to the above regions, the same is probably valid for a generic record for mainland Italy (Zhantiev 2011a).

#### Remarks.

First detailed records from Apulia. About Sicily, Ragusa (1892) wrote *Ne posseggo vari esemplari [= I have several specimens]* without giving the collecting sites, so the sole known precise Sicilian localities were the Pantelleria and Lipari Islands (Liebmann 1962, Lo Cascio et al. 2006); the above new site is located in one of the most important forested areas of western Sicily, a zone of great value for environmental conservation (cf. Sabella and Sparacio 2004, Mason et al. 2006, La Mantia et al. 2010, Falci et al. 2012). Luigioni (1923a) also recorded *Anthrenus (Anthrenus) delicatus* from the environs of Rome; this record was overlooked by Nardi (1997). As stated by Audisio et al. (1995: 16), the Italian distribution of this species should be verified, because it was considered for a long time as a variety or aberration of *Anthrenus (Anthrenus) pimpinellae*. Moreover, the colour of elytral fasciae in four of the above Apulian specimens are visually very similar to those of *Anthrenus (Anthrenus) mroczkowskii* Kalík, 1954, nevertheless their identification was established by the examination of the structure of antennae, 9th sternite and male genitalia (cf. Kadej 2005, Kadej et al. 2007). In this framework, it will be better to revise the specimens of *Anthrenus (Anthrenus) mroczkowskii* recorded so far from Italy (Angelini 1986, as *Anthrenus pimpinellae mroczkowskii*, Angelini and Montemurro 1986, as *Anthrenus pimpinellae mroczkowskii*). The distribution of *Anthrenus (Anthrenus) mroczkowskii* also includes the following countries: Albania, Bosnia and Herzegovina, Bulgaria, Corsica, Crete, Croatia, Greece, Slovenia, European Turkey and Algeria (introduced) (Háva 2007, as *Anthrenus (Anthrenus) pimpinellae mroczkowskii*, Kadej et al. 2007).

### Anthrenus (Anthrenus) munroi

Hinton, 1943

http://species-id.net/wiki/Anthrenus_munroi

Anthrenus munroi Hint.: Angelini 1986: 73; Angelini and Montemurro 1986: 573; Angelini 1987: 37.
Anthrenus (Anthrenus) munroi Hinton, 1943: Háva and Nardi 2011: 425.

#### Material examined.

Tuscany: [Livorno prov.], Is. [= Isola = Island] d’Elba, dint. Marina di Campo, 15–17.VI.1999, Abbazzi, Bartolozzi, Lo Cascio & Sforzi leg., 1 ♂ (MZUF).

#### Chorotype.

Mediterranean (eastwards as far as Ukraine, Turkey, Syria, Lebanon, Jordan, and Israel), with extension to Hungary and Portugal (cf. Kadej et al. 2007, Háva and Nardi 2011).

#### Italian distribution.

Apulia, Basilicata, Calabria (Angelini 1986, Angelini and Montemurro 1986, Angelini 1987) and Sardinia (Háva and Nardi 2011).

#### Remarks.

First record for central Italy; this new site is the northernmost in Italy. The species also occurs in the facing Corsica (cf. Háva and Nardi 2011).

*Anthrenus (Anthrenus) pimpinellae pimpinellae* (Fabricius, 1775) and *Anthrenus (Nathrenus) verbasci* (Linnaeus, 1767) were the sole congeneric species previously recorded from Elba Island (Holdhaus 1923, as *Anthrenus pimpinellae* F. and *Anthrenus verbasci* L.; Luigioni 1929, Porta 1929, both as *Anthrenus (Anthrenus) pimpinellae* Fabr. and *Anthrenus (Nathrenus) verbasci* Lin.)

### Anthrenus (Anthrenus) pimpinellae pimpinellae

(Fabricius, 1775)

http://species-id.net/wiki/Anthrenus_pimpinellae_pimpinellae

Anthrenus pimpinellae F.: Ragusa 1892: 206; Bertolini 1904: 59; Holdhaus 1911: 445; Panganetti-Hummler 1918: 87; Holdhaus 1923: 101; Luigioni 1923b: 4; Marcuzzi 1985: 8.
Anthrenus (Anthrenus) pimpinellae Fabr.: Luigioni 1929: 540; Porta 1929: 306.
Anthrenus (Anthrenus) pimpinellae pimpinellae (Fabricius, 1775): Háva and Nardi 2004: 120; Háva and Nardi 2011: 426.
Anthrenus (Anthrenus) pimpinellae (Fabricius, 1775) s.l.: Lo Cascio et al. 2006: 322.

#### Material examined.

Apulia: Foggia prov., [GNP,] Promontorio del Gargano, Vieste, SP 89 [= Strada Provinciale n. 89 = Provincial Road n. 89], Casa Cupari dint., 354 m, 33T 5920001 4630150, abandoned quarry, sn on grasses, 29.V.2010, PC, Prog. Foresta Umbra, 4 ex (CNBFVR).

#### Chorotype.

Cosmopolitan (cf. Háva and Nardi 2011).

#### Italian distribution.

All of Italy and Sicily (Bertolini 1904, Luigioni 1929, Porta 1929; Háva and Nardi 2011). This species is also recorded from some minor islands: Elba (Holdhaus 1923), Capri (Luigioni 1923b, 1929, Porta 1929), Lampedusa (Ragusa 1892, Luigioni 1929) and Vulcano (Lo Cascio et al. 2006).

#### Remarks.

Species common and widespread in Italy but from the Gargano National Park (cf. Angelini 1987) was recorded only by Holdhaus (1911); it is also known from other Apulian localities (Panganetti-Hummler 1918, Marcuzzi 1985).

### Anthrenus (Nathrenus) signatus

Erichson, 1846

http://species-id.net/wiki/Anthrenus_signatus

Anthrenus (Nathrenus) signatus Erichson, 1846: Háva and Nardi 2011: 428.

#### Material examined.

Basilicata: Potenza prov., Pignola, Ris. [= Riserva = Reserve] WWF, L. [= Lago = Lake] Pignola, 700 m, 21.VI.1992, FA, 1 ♂ (JHAC).

#### Chorotype.

European (cf. Háva and Nardi 2011).

#### Italian distribution.

South Tyrol, Venetia, Venezia Giulia, Latium?, Apulia, Basilicata and Sicily (cf. Háva and Nardi 2011).

#### Remarks.

This species was recorded in Basilicata region from the Pollino National Park (Angelini 1986) only; the above specimen comes from a nature reserve, from which a sole congeneric species, *Anthrenus (Nathrenus) verbasci*, was known so far (Angelini 1996a, 1998, both as *Anthrenus verbasci*).

*Anthrenus signatus* ab. *bielawskii* Mroczkowski, 1958 from Bulgaria (Mroczkowski 1958: 6, 1968: 145) was recently ignored (Háva 2007, 2008, 2011a), since according to the ICZN (1999, art. 45.5 and 45.6.2), it is an infrasubspecific and unavailable name.

### Globicornis (Globicornis) breviclavis

(Reitter, 1878)

http://species-id.net/wiki/Globicornis_breviclavis

Hadrotoma breviclavis Reitter: Baudi 1890: 101.
Hadrotoma breviclavis Reitt.: Bertolini 1904: 59.
Globicornis (Globicornis) breviclavis Reitt.: Ganglbauer 1904: 31; Reitter 1906: 379; Dalla Torre 1911: 63; Winkler 1926: 679; Luigioni 1929: 539; Porta 1929: 304.
Globicornis (Globicornis) breviclavis (Reitter, 1878): Audisio et al. 1995: 13, 16.

#### Material examined.

None.

#### Chorotype.

Endemic to the Near East: Armenia, Bulgaria, “Caucasus”, Georgia, European Turkey, Ukraine, and Southern European Russia (Adygeya, Krasnodar kraj) (Háva 2007, Háva and Legalov 2010, Zhantiev 2011a).

#### Italian distribution.

Piedmont: Graian and Pennine Alps (Baudi 1890, Luigioni 1929, Porta 1929); Piedmont (without further details) (Bertolini 1904, Reitter 1906); Piedmont? (Dalla Torre 1911, Winkler 1926, Audisio et al. 1995). Liguria: Ligurian Apennines (Luigioni 1929); Liguria? (Audisio et al. 1995).

#### Remarks.

This species described from Caucasus (Reitter 1878, as *Hadrotoma breviclavis*) is excluded here from the Italian fauna. Ganglbauer (1904) listed the paper by Baudi (1890), while Dalla Torre (1911) referred to Ganglbauer (1904). The Italian records were ignored by Mroczkowski (1968: 114), who recorded this species only from the Caucasus. No specimens of this species are present in the P. Luigioni collection (MCZR) (A. Zilli, pers. comm. 2013). The above Italian records probably concern the similar *Globicornis (Globicornis) luckowi* Herrmann, Háva & Kadej, 2011 which is known only from southern Switzerland, Piedmont and Liguria (Herrmann et al. 2011, Háva et al. 2013). Nevertheless, as discussed for *Attagenus (Attagenus) simonis*, an escatomediterranean distributional pattern (Zilli 2000) could explain the occurrence of *Globicornis (Globicornis) breviclavis* in Italy.

### Globicornis (Globicornis) fasciata

(Fairmaire & Brisout de Barneville, 1859)

http://species-id.net/wiki/Globicornis_fasciata

Hadrotoma fasciata Fairm.: Bertolini 1904: 59; Luigioni 1923a: 135.
Globicornis fasciata Fair.: Vitale 1904: 75.
Globicornis (Globicornis) fasciata Frm. [18]59: Winkler 1926: 679.
Globicornis (Globicornis) fasciata Fairm.: Luigioni 1929: 539; Porta 1929: 304.
Globicornis fasciatus [sic!] Fairm.: Horion 1955: 208.
Globicornis (Globicornis) fasciata (Fairmaire, 1859): Mroczkowski 1968: 114; Audisio et al. 1995: 13, 16; Zhantiev 2011a.
Globicornis (Globicornis) fasciata (Fairmaire & Brisout, 1859): Háva 2003: 109; Háva and Nardi 2011: 429.
Globicornis (Globicornis) fasciata (Fairmaire & Brisout de Barneville, 1859): Háva 2007: 315.

#### Material examined.

Basilicata: Potenza prov., Parco Naz. del Pollino [= Pollino National Park], Casa del Conte dint., tra [= between] Acqua Tremola e [= and] Piani di S. Francesco, 1100–1500 m, 9.VII.2002, sn, GC, 3 ex (PCOP).

#### Chorotype.

European (Belgium, Denmark, France, Germany, Italy, Spain, Sweden, Switzerland) with extension southward to Tunisia (cf. Háva et al. 2010, Háva and Nardi 2011).

#### Italian distribution.

Piedmont (Porta 1929, Horion 1955), Tuscany (Luigioni 1929, Horion 1955), Latium (Luigioni 1923a, 1929, Porta 1929, Horion 1955), Sicily (Vitale 1904) and Sardinia (Bertolini 1904, Winkler 1926, Luigioni 1929, Porta 1929, Horion 1955, Mroczkowski 1968, Audisio et al. 1995, Háva 2003, 2007, Zhantiev 2011a).

The records for northern and peninsular Italy (Audisio et al. 1995) as those generic for Italy (Háva 2003) refers to above regions; the same is probably valid for a generic record for mainland Italy (Zhantiev 2011a).

#### Remarks.

First record from southern Italy. Five species of this genus are now recorded from the Pollino National Park (cf. Angelini 1986).

The records for Piedmont (Porta 1929, Horion 1955) and those generic for northern Italy (Audisio et al. 1995: 13) refers to the Maritime Alps (Audisio et al. 1995: 16). The species also occurs in a neighbouring French department (Sainte-Claire Deville 1937: 252, as *Globicornis fasciata* Fairm.). The record from Sicily (Vitale 1904) was omitted by subsequent authors (*cf*. Luigioni 1929, Audisio et al. 1995, Zhantiev 2011a), and thus, it should be confirmed.

In the literature (see also above), the authorship of this species was often attributed only to Fairmaire.

### Globicornis (Hadrotoma) sulcata

(C.N.F. Brisout de Barneville, 1866)

http://species-id.net/wiki/Globicornis_sulcata

Globicornis ? sulcata (C. Brisout de Barneville, 1866): Angelini 1986: 74.
Globicornis (Hadrotoma) ? sulcata (C. Brisout de Barneville, 1866): Audisio et al. 1995: 16.
Globicornis (Hadrotoma) sulcata (C. Brisout de Barneville, 1866): Audisio et al. 1995: 13.

#### Material examined.

Abruzzi: Parco Naz. [= National Park], L‘Aquila prov., Pescasseroli, 1300 m, 1.IV.2003, FA, 1 ♀ (JHAC). Basilicata: Potenza prov., Moliterno, 500 m, 11.V.2003, FA, 1 ♀ (JHAC); Potenza prov., Ruoti, 29.IX.1996, *Abies*–*Quercus*, FA, 1 ♂ (JHAC). Campania: Avellino prov., Cilento, Novi Velia, M. [= Mount] Sacro, 1900 m, 7.XII.1996, *Fagus*, FA, 1 ♂ (JHAC). Sicily: Palermo prov., Ficuzza, 700 m, 1–4.V.2000, bosco leccio [= ilex wood], FA, 1 ♂ (JHAC); Palermo prov., Bosco della Ficuzza, 27.V.2006, M. Sarovec leg., 1 ♂, 4 ♀♀ (JHAC).

#### Chorotype.

W-Mediterranean: France, Italy and Spain.

#### Italian distribution.

Basilicata (Angelini 1987, Audisio et al. 1995).

#### Remarks.

First records from central Italy (Abruzzi), Campania and Sicily. In the Sicilian site, it was also collected *Anthrenus (Anthrenus) delicatus* (see above).

### Trogoderma angustum

(Solier, 1849)

http://species-id.net/wiki/Trogoderma_angustum

Trogoderma angustum (Solier, 1849): Háva 2007: 319; Zhantiev 2011a.

#### Material examined.

None.

#### Chorotype.

Subcosmopolitan (cf. Háva 2007). This species is also recorded from Switzerland (Kenis 2005); this country must be added to the European distributions recently summarized by Háva (2007) and by Zhantiev (2011a).

#### Italian distribution.

Italy (without further details) (Háva 2007). Italian mainland (Zhantiev 2011a).

#### Remarks.

The above generic Italian record by Háva (2007) was based on a personal communication from V. Kalík, who identified Italian material; unfortunately, no further details are available (Háva, unpublished data).

### Trogoderma inclusum

LeConte, 1854

http://species-id.net/wiki/Trogoderma_inclusum

Trogoderma meridionalis Kraatz, 1858: 146.
Trogoderma meridionalis Kraatz: Mulsant and Rey 1868: 132.
Trogoderma meridionale [sic!] Kraatz: Bertolini 1874: 99.
Trogoderma versicolor Creutz. [= (Creutzer, 1799)] var. *meridionale* [sic!] Kr.: Ragusa 1892: 205; Porta 1929: 305.
Trogoderma versicolor Creut. v. *meridionale* [sic!] K.: Bertolini 1904: 59.
Trogoderma versicolor Creutz. a. *meridionale* [sic!] Kr.: Luigioni 1929: 540.
Trogoderma versicolor meridionale [sic!] Kraatz, 1858: Mroczkowski 1968: 107.
Trogoderma inclusum Le Conte: Dal Monte 1972: 102.
Trogoderma ? inclusum Lec.: Angelini and Montemurro 1986: 573.
Trogoderma inclusum : Nardi R. et al. 1993: 623, fig. 9.
Trogoderma [?] inclusum J.L. Leconte, 1854: Audisio et al. 1995: 13, 16.
Trogoderma inclusum Leconte: Nicoli Aldini 1999: 8.
Trogoderma inclusum LeConte, 1854: Háva 2007: 319; Háva and Nardi 2011: 432; Zhantiev 2011a.

#### Material examined.

Apulia: Lecce prov., Salento, Torre S. Giovanni dint., 1–7.VII.2002, PC GC, sn, 1 ex (PCOP). Venetia: Verona prov., Verona, [Fraz.] Cancello, [about 500 m], 22.VI.1986, sn, PC, 1 ex (PCOP).

#### Chorotype.

Cosmopolitan (cf. Háva and Herrmann 2011, Háva and Nardi 2011, Háva et al. 2011).

#### Italian distribution.

Lombardy (Nicoli Aldini 1999, Háva and Nardi 2011), Liguria (Luigioni 1929), Emilia-Romagna (Nicoli Aldini 1999), Latium (Nardi R. et al. 1993, Nicoli Aldini 1999), Basilicata [?] (Angelini and Montemurro 1986, Audisio et al. 1995), Sicily (Kraatz 1858, Mulsant and Rey 1868, Ragusa 1892, Bertolini 1904, Porta 1929, Mroczkowski 1968) and Sardinia (cf. Háva and Nardi 2011).

The first Italian record of this species was provided by Kraatz (1858) who recorded it from Sicily (without a precise locality), while some authors (Bertolini 1874, Dal Monte 1972, Háva 2007) and Zhantiev (2011a) recorded it from Italy and mainland Italy, respectively, without further details. Moreover, Nicoli Aldini (1999: 8) also recorded it from *alcune città del Nord-Italia [= some towns of Northern Italy]* but without mentioning the regions.

#### Remarks.

In Italy, this species and *Trogoderma versicolor* (Creutzer, 1799) have often been confused (cf. Nicoli Aldini 2003b, Háva and Nardi 2011); old Italian records in the literature must be thus considered with caution. *Trogoderma inclusum* is recorded here for the first time from Apulia and Venetia. This species is a stored product pest; it also develops in nests of social aculeate Hymenoptera, and is a predator of egg masses of *Lymantria dispar* (Linnaeus, 1758) (Lepidoptera, Erebidae, Lymantriinae) (cf. Háva and Nardi 2011).

### Literature records

The old Sicilian records of *Attagenus (Attagenus) trifasciatus* (Fabricius, 1787) and *Trogoderma glabrum* (Herbst, 1783) (Ghiliani 1842) were ignored (or overlooked?) by subsequent authors and are thus considered as doubtful here (Table 1).

*Thorictus mauritanicus* Lucas, 1846 and *Thorictus stricticollis* Kraatz, 1859 were both recorded from “Italy” by Háva (2003, 2007) and from “Italian mainland” by Zhantiev (2011a). Háva’s (2003, 2007) records of *Thorictus mauritanicus* refer only to Sardinia and Sicily, while those of *Thorictus stricticollis* (Háva 2003, 2007) refer only to Sardinia (Háva, unpublished data). In this framework, the records of both species for mainland Italy (Zhantiev 2011a) are considered erroneous.

*Thorictus sicilianus* John, 1965 (= *Thorictus sicilianus* John, 1966) from Sicily and Morocco (John 1965, 1966, Audisio et al. 1995, Löbl 2007) was recently recognized as a junior synonym of *Thorictus grandicollis grandicollis* Germar, 1842 (Háva 2013); *Attagenus picipennis* Pic, 1894 from Sicily (Audisio et al. 1995) is a junior synonym of *Attagenus (Attagenus) brunneus* Faldermann, 1835 (Háva 2007: 57); *Anthrenus (Attagenus) scrophulariae suecius* Palm, 1940 that was recorded from southern Italy and Sicily (Audisio et al. 1995), was recently listed by Háva (2007: 313) as a junior synonym of *Anthrenus (Anthrenus) scrophulariae scrophulariae* (Linnaeus, 1758), but for an error this new synonym was not formally established (cf. Háva 2007: 57). These three taxa are thus excluded from the new checklist.

***Dermestes (Dermestinus) gyllenhalii gyllenhalii*** Laporte de Castelnau, 1840

*Dermestes ? atomarius* Erichson, 1846: Sapuppo 2002: 189.

Si? (Sapuppo 2002).

***Dermestes (Dermestinus) laniarius laniarius*** Illiger, 1801

*Dermestes laniarius* Illig.: Gulli 1961: 4.

*Dermestes laniarius* Illiger, 1801: Sapuppo 2002: 190.

Si (Gulli 1961, Sapuppo 2002).

***Thorictus grandicollis grandicollis*** Germar, 1842

*Thorictus grandicollis* Germ.: Cerruti 1954: 111.

N (Cerruti 1954).

***Attagenus (Attagenus) cyphonoides*** Reitter, 1881

*Attagenus alfierii* Pic[, 1910]: Trematerra 1987: 16.

*Attagenus (Attagenus) cyphonoides* Reitter, 1881: Háva and Nardi 2004: 121, Háva 2007: 308.

*Attagenus (Attagenus) cyphonoides* Reitter, 1881: Zhantiev 2011a.

S (Háva and Nardi 2004). Italy (Trematerra 1987, Háva 2007). Italian mainland (Zhantiev 2011a).

***Attagenus (Attagenus) fasciatus*** (Thunberg, 1795)

*Attagenus fasciatus* (Thunberg, 1795): Nicoli Aldini 2003a 155–156, Nicoli Aldini 2003b: 123–125, Nicoli Aldini 2004: 983, 985, Zhantiev 2011a.

*Attagenus (Attagenus) fasciatus* (Thunberg, 1795): Háva and Nardi 2004: 121, Háva 2007: 308.

N (Nicoli Aldini 2003a, 2003b, 2004), S (Háva and Nardi 2004). Italy (Háva 2007). Italian mainland (Zhantiev 2011a).

***Attagenus (Attagenus) pantherinus*** (Ahrens, 1814)

*Attagenus pantherinus*: Henry 1938: 66.

*Attagenus (Attagenus) pantherinus* (Ahrens, 1814): Háva 2007: 309, Háva and Nardi 2007: 121.

*Attagenus pantherinus* (Ahrens, 1814): Zhantiev 2011a.

N (Henry 1938, Háva and Nardi 2007), Sa (Zhantiev 2011a). Italy (Háva 2007).

***Attagenus (Attagenus) rossii*** Ganglbauer, 1904

*Dermestes bifasciatus* Rossi, 1794: 79.

*Attagenus bifasciatus* Rossi: Rottenberg 1870: 238, Bertolini 1874: 99, Steck 1887: 181, Bertolini 1904: 58.

*Attagenus bifasciatus*: Failla-Tedaldi 1887: 158.

*Attagenus (Lanorus) bifasciatus* Rossi: Ragusa 1892: 204.

*Attagenus (Lanorus) bifasciatus* Oliv. [= (A.G. Olivier, 1790)] a. *Rossii* Ganglb.: Luigioni 1929: 538.

*Attagenus (Lanorus) bifasciatus* Oliv. v. *Rossii* Ganglb.: Porta 1929: 302.

*Attagenus bifasciatus* Oliv. var. *Rossii* Ganglb.: Gulli 1961: 4.

*Attagenus bifasciatus* (Olivier, 1790) var. *Rossii* Ganglbauer, 1904: Sapuppo 2002: 187.

*Attagenus (Attagenus) rossii* Ganglbauer, 1904: Háva 2007: 309, Háva and Nardi 2007: 123, Háva and Nardi 2011: 422.

*Attagenus rossii* Ganglbauer, 1904: Zhantiev 2011a.

*Attagenus rossii* Ganglbauer, 1804 [sic!]: FEI 2012.

S (Rossi 1794, Luigioni 1929, Porta 1929, Háva and Nardi 2007, 2011), Si (Rottenberg 1870, Bertolini 1874, Failla-Tedaldi 1887, Steck 1887, Ragusa 1892, Bertolini 1904, Luigioni 1929, Porta 1929, Gulli 1961, Sapuppo 2002, Háva and Nardi 2007, Zhantiev 2011a, FEI 2012), Sa (cf. Háva and Nardi 2011). Italy (Háva 2007). Italian mainland (Zhantiev 2011a).

***Attagenus (Attagenus) trifasciatus*** (Fabricius, 1787)

*Attagenus trifasciatus* Fabr.: Ghiliani 1842: 33.

*Attagenus ? trifasciatus* (Fabricius, 1787): Sapuppo 2002: 189.

Si? (Ghiliani 1842, Sapuppo 2002).

***Anthrenocerus australis*** (Hope, 1843)

*Anthrenocerus australis* (Hope, 1843): Herrmann and Háva 2007: 5, Háva 2007: 315, Zhantiev 2011a.

N (Herrmann and Háva 2007). Italy (Háva 2007). Italian mainland (Zhantiev 2011a).

***Globicornis (Globicornis) luckowi*** Herrmann, Háva & Kadej, 2011

*Globicornis (Globicornis) luckowi* Herrmann, Háva & Kadej, 2011: Háva et al. 2013: 138.

N (Háva et al. 2013).

***Globicornis (Hadrotoma) corticalis*** (Eichhoff, 1863)

*Globicornis corticalis* (Eichhoff, 1863): Biscaccianti et al. 2008: 50.

S (Biscaccianti et al. 2008).

***Reesa vespulae*** (Milliron, 1939)

*Reesa vespulae* (Milliron, 1939): Nicoli Aldini 2003a: 156, 159, Nicoli Aldini 2003b: 123, 127, Nicoli Aldini 2004: 983, 987.

N (Nicoli Aldini 2003a, 2003b, 2004).

***Trogoderma glabrum*** (Herbst, 1783)

*Trogoderma elongatulum* Fabr. [= (Fabricius, 1801)]: Ghiliani 1842: 33.

Si? (Ghiliani 1842).

***Trogoderma variabile*** Ballion, 1878

*Trogoderma variabile* Ballion, 1878: Nicoli Aldini 1998: 115, Háva 2003: 141, Háva 2007: 320.

*Trogoderma variabile* Ballion: Nicoli Aldini 2003a: 156, Nicoli Aldini 2003b: 126–127.

N (Nicoli Aldini 1998, 2003a, 2003b), Sa (Zhantiev 2011a). Italy (Háva 2003, 2007). Italian mainland (Zhantiev 2011a).

### A new checklist of the Dermestidae of Italy

The present-day dermestid fauna of Italy is summarized in the checklist below (Table 1).

The following abbreviations are used: AFR = Afrotropical; ALAP = Alpino-Apenninic endemic; ALPC = Central-Alpine endemic; APPE = Apenninic endemic; AUST = Australian; CAE = Centralasiatic-European; CEM = Centralasiatic-Europeo-Mediterranean; CEU = Central European; COS = Cosmopolitan; EME = E-Mediterranean; EUM = Europeo-Mediterranean; EUR = European; IT = Italy and/or Italian mainland without further details; (i) = introduced to Europe; MED = Mediterranean; N = northern Italy (Trentino-Alto Adige, Venetia, Friuli-Venezia Giulia, Lombardy, Piedmont, Aosta Valley, Liguria and Emilia-Romagna); NAF = N-African; OLA = Holarctic; PAL = Palearctic; S = peninsular Italy (the remainig of continental Italy); Sa = Sardinia and small circum-Sardinian islands; SCO = Subcosmopolitan; SEU = S-European; Si = Sicily and small circum-Sicilian islands; SICI = Sicilian endemic; SIE = Sibero-European; TEM = Turano-Europeo-Mediterranean; TUE = Turano-European; TUM = Turano-Mediterranean; WME = W-Mediterranean.

## Discussion

The updated checklist of Italian Dermestidae includes 95 species, moreover *Anthrenus (Anthrenus) pimpinellae* occurs with two subspecies, but the taxonomic status of *Anthrenus (Anthrenus) pimpinellae isabellinus* needs to be clarified (cf. Háva and Nardi 2004). This total number is very probably still provisional since this family has been little studied in Italy, and the occurrence of further species recently described (e.g. Zhantiev 2001) or resurrected is possible. The previous checklist (Audisio et al. 1995), included only 81 species. Considering the four adopted geographic divisions, the relatively high species richness in the peninsular region (67) is unsurprising (cf. Minelli et al. 2002).

Some of the above records come from the following two protected areas of southern peninsular Italy: the Gargano National Park (Apulia) and the Pollino National Park (Basilicata and Calabria). 24 species of Dermestidae are now known from the former Park, while 23 species are now known from the Pollino National Park, but only 15 species are common to both (cf. Holdhaus 1911, Panganetti-Hummler 1918, Angelini 1987, 1996b, Háva and Nardi 2004). 24 and 23 species represent 37% and 34% of those currently recorded with certainty from peninsular Italy. These values confirm the importance of these Parks for insects (cf. Holdhaus 1911, Palm 1939, Grandi 1956, Angelini 1986, 1987, Rusek and Stumpp 1988, Agostini and Scali 1989, Rivosecchi 1989, Merz 1993, Angelini 1996b, Ruffo and Stoch 2006) and for biodiversity conservation.

## Supplementary Material

XML Treatment for Dermestes (Dermestes) peruvianus

XML Treatment for Dermestes (Dermestinus) carnivorus

XML Treatment for Dermestes (Dermestes) vorax

XML Treatment for Dermestes (Dermestinus) hankae

XML Treatment for Dermestes (Dermestinus) intermedius intermedius

XML Treatment for Dermestes (Dermestinus) szekessyi

XML Treatment for Dermestes (Montandonia) olivieri

XML Treatment for Thorictus pilosus

XML Treatment for Thorictus wasmanni

XML Treatment for Attagenus (Attagenus) calabricus

XML Treatment for Attagenus (Attagenus) lobatus

XML Treatment for Attagenus (Attagenus) pilosissimus

XML Treatment for Attagenus (Attagenus) silvaticus

XML Treatment for Attagenus (Attagenus) simonis

XML Treatment for Attagenus (Attagenus) tigrinus pulcher

XML Treatment for Anthrenus (Anthrenops) coloratus

XML Treatment for Anthrenus (Anthrenus) angustefasciatus

XML Treatment for Anthrenus (Anthrenus) delicatus

XML Treatment for Anthrenus (Anthrenus) munroi

XML Treatment for Anthrenus (Anthrenus) pimpinellae pimpinellae

XML Treatment for Anthrenus (Nathrenus) signatus

XML Treatment for Globicornis (Globicornis) breviclavis

XML Treatment for Globicornis (Globicornis) fasciata

XML Treatment for Globicornis (Hadrotoma) sulcata

XML Treatment for Trogoderma angustum

XML Treatment for Trogoderma inclusum
